# Structural and functional imaging markers for susceptibility to psychosis

**DOI:** 10.1038/s41380-020-0679-7

**Published:** 2020-02-17

**Authors:** Christina Andreou, Stefan Borgwardt

**Affiliations:** 1 Department of Psychiatry and Psychotherapy, University of Lübeck, Lübeck, Germany; 2 Department of Psychiatry (UPK), University of Basel, Basel, Switzerland

**Keywords:** Schizophrenia, Neuroscience

## Abstract

The introduction of clinical criteria for the operationalization of psychosis high risk provided a basis for early detection and treatment of vulnerable individuals. However, about two-thirds of people meeting clinical high-risk (CHR) criteria will never develop a psychotic disorder. In the effort to increase prognostic precision, structural and functional neuroimaging have received growing attention as a potentially useful resource in the prediction of psychotic transition in CHR patients. The present review summarizes current research on neuroimaging biomarkers in the CHR state, with a particular focus on their prognostic utility and limitations. Large, multimodal/multicenter studies are warranted to address issues important for clinical applicability such as generalizability and replicability, standardization of clinical definitions and neuroimaging methods, and consideration of contextual factors (e.g., age, comorbidity).

## Introduction

Psychotic disorders pose a major challenge for psychiatric practice and public health, affecting several aspects of functioning and quality of life and being among the leading causes of disease burden worldwide [1]. Antipsychotic medication efficacy remains stable at a moderate effect size despite the introduction of several new antipsychotics [2], and recovery rates have not improved in the past 70 years [3].

In the 1990s, a paradigm shift in psychosis conceptualization and research led to an increased focus on the early stages of psychotic disorders, including the period before the onset of overt symptoms [4]. The introduction of clinical criteria for the operationalization of psychosis high risk provided a basis for early detection and treatment of vulnerable individuals, with the ultimate goal to improve outcomes by delaying or preventing the onset of psychotic disorders, and/or ensuring timely treatment.

Currently, diagnosis of a clinical high-risk state is usually made based on ultra-high-risk (UHR) criteria, requiring the presence of either (a) positive symptoms that are typical of psychotic disorders but of subthreshold severity (attenuated psychotic symptoms; APS) or duration (brief limited intermittent psychotic symptoms; BLIPS), or (b) genetic high risk accompanied by functional decline (GRD) [5, 6]. A complementary approach focuses on basic symptoms [6], i.e., subjective changes in perception, cognition, and language that are conceptually less closely related to psychotic symptoms and have been suggested to indicate an earlier risk stage than UHR criteria [7].

Individuals meeting high-risk criteria are at substantially higher risk for developing a psychotic disorder compared with the general population [4, 8]. Most transitions occur within 2–3 years from initial assessment [4, 8, 9] and result in a schizophrenia spectrum disorder [10]. However, with a sensitivity of 96% but only modest specificity of 47% [11], clinical high-risk criteria are more useful for excluding, rather than predicting, a future outcome of psychosis [12]. About two-thirds of individuals meeting high-risk criteria will not develop a psychotic disorder [4]; accordingly, there is substantial criticism to the psychosis high-risk paradigm, pointing out issues such as the lack of prognostic precision, specificity for psychosis, and the potential for unnecessary (self-)stigmatization and unindicated antipsychotic drug treatment in individuals receiving a diagnosis of high psychosis risk [13–15].

Given the above limitations of clinical high-risk criteria, there have been intensive efforts to improve specificity and predictive accuracy with respect to transition to psychosis using individual symptom profiles [16, 17], demographic [18], or cognitive variables [19]. In this context, structural and functional neuroimaging have received growing attention as a potentially useful resource in the prediction of psychotic transitions, as psychotic disorders are linked to several well-established abnormalities of brain structure and function. It is expected that neuroimaging can complement clinical judgment by providing standardized, objective biomarkers for predicting transition in high-risk individuals. The present review summarizes current research on neuroimaging biomarkers with a particular focus on their prognostic utility and limitations.

## Structural neuroimaging

### Gray matter

Gray matter abnormalities are probably the best studied neuroimaging biomarker in high-risk patients so far. Early MRI investigations used manual tracing to investigate regions of interest assumed to be affected in psychotic disorders. In contrast, more recent studies have mainly relied on automated approaches, which have the advantage of allowing group comparisons at the whole-brain level rather than specific regions [20, 21], thus being better suited for investigation of the complex neuroanatomical abnormalities expected to occur in patients with psychotic disorders. The most common such approach is voxel-based morphometry (VBM), which assesses between-group differences in regional volume or tissue composition based on estimates of tissue probability on a voxel-wise basis, as opposed to predefined anatomical borders [22]. It should be noted, though, that VBM is very sensitive to image registration procedures, and there is an ongoing debate on whether apparent volumetric changes may in fact reflect changes in position in some cases, especially given sulcal patterning abnormalities in patients with schizophrenia [23, 24]. Volumetric approaches can be therefore complemented by surface-based techniques that assess measures such as cortical thickness [25], surface area [26] or gyrification (i.e., cortical folding) [27]. Evidence suggests that the characteristic convolutions of the cortical surface emerge through the axonal tension produced by neuronal connections during brain development in the 2nd and 3rd trimester of pregnancy [28]. Interestingly, different surface-based measures are differentially affected by genes and neurodevelopmental stage [29, 30]. Therefore, these additional measures may additionally contribute to a more refined understanding of the neurodevelopmental abnormalities postulated to underlie psychotic disorders.

Several volumetric differences in gray matter have been reported in high-risk patients compared with healthy controls, the most consistent being volume reductions in hippocampal/parahippocampal areas, cingulate cortex, as well as the medial and lateral frontal cortex and medial parietal cortex [21, 31–33]. Studies comparing high-risk subjects with (CHR-T) and without (CHR-NT) later transition to psychosis indicates that some of these volume reductions might be relevant for the prediction of future psychotic transition. Although there is some variability in findings, certain areas have been repeatedly been shown to be associated with later psychosis in high-risk subjects. These include the anterior cingulate [34–36], frontal cortex [35, 37, 38], temporal cortex [34, 35, 37, 39], parietal cortex [38, 39], cerebellum [35, 37], and insular cortex [34, 40]. Moreover, CHR-T show larger pituitary volumes than CHR-NT [41]. Somewhat surprisingly given the prominent place of hippocampal dysfunction in theories of schizophrenia pathophysiology [42], findings are less consistent with respect to hippocampal areas and psychotic transition: three studies reported reduced hippocampal volume in CHR-T compared with CHR-NT [35, 43, 44]; however, others failed to find any differences in hippocampal volume [45–49] or cortical thickness [50] between the two patient groups. Beyond volumetric differences, a recent study [51] reported aberrant structural covariance patterns in the salience, executive control, auditory and motor networks in CHR-T compared with CHR-NT; although differences were subtle, they are consistent with reports of functional connectivity abnormalities in CHR patient populations (see below).

Beyond static comparisons, evidence suggests that the dynamic of changes in brain morphology over time might also be informative for predicting transition in CHR patients. CHR-T have been reported to exhibit greater longitudinal volume reduction and/or cortical thinning in frontal areas, including orbitofrontal, superior frontal, middle frontal, and prefrontal cortices [35, 37, 52–54]; temporal areas such as the inferior [37] and middle temporal cortex [55], and the fusiform and parahippocampal cortex [35]; the cingulate cortex [35, 55, 56]; the cerebellum [35, 37]; the medial and superior parietal lobes [37] and precuneus [55]; and the insular cortex [40]. These findings appear to be independent of antipsychotic medication treatment, as they did not differ between UHR patients with and without antipsychotic drug exposure [54], and are consistent with the notion of neurodevelopmental abnormalities leading to the emergence of psychotic disorders; however, they may also result from a range of other factors such as substance abuse, stress due to increased symptom load, or pharmacological treatment [21, 57]. The effects of such confounding factors have not yet been systematically assessed.

### White matter

White matter density and regional distribution are age-sensitive, and its development continues well into late adolescence and beyond [58]. Therefore, white matter studies in CHR patients might help elucidate abnormal neurodevelopmental trajectories in psychotic disorders.

The most widely used technique to assess white matter integrity is diffusion tensor imaging (DTI), which measures the diffusion of water molecules through tissues using T2-weighted images in diverse directions. Although volume-based measures of white matter integrity similar to those for gray matter are available, track-based statistics are much more popular due to their superior accuracy [59]. The most common DTI-derived marker of white tract integrity is fractional anisotropy (FA), which provides an estimate of the net directionality of diffusion independent of fiber orientation. FA decreases reflect changes in neural fiber density, axon diameter, and myelination [60–62] but do not allow inferences about the nature of alterations (e.g., axonal degeneration, demyelination, or simply low signal-to-noise ratio) [63]. Such information is provided by indices of diffusion magnitude, i.e., axial (AD), radial (RD), and mean (MD) diffusivity, which are therefore important complementary measures to FA. For instance, axonal degeneration may be expressed in reduction in FA and AD, along with an increase in MD and RD, whereas demyelination displays a similar pattern, but without a change in AD [64].

FA is generally lowered in multiple brain regions in patients with schizophrenia [58]. CHR have been reported to display reduced whole white matter volume [65] as well as decreased FA the inferior fronto-occipital fasciculus (IFOF) and the inferior longitudinal fasciculus connecting the anterior temporal to the occipital lobe [66, 67], the superior longitudinal fasciculus that connects the frontal to the superior temporal, occipital and cerebellar cortices through the inferior parietal lobe and the supermarginal gyrus [66, 68, 69], and the corpus callosum [66, 69, 70]. Abnormal thalamocortical connectivity, considered to be one of the core pathologies in psychotic disorders, has also been reported in CHR patients, particularly affecting thalamus-orbitofrontal [71] and frontal-striatal-thalamic connections [72]; reduction in these thalamocortical tracts correlated with symptom severity and global social functioning, respectively [71, 72].

Unfortunately, there are few and inconsistent findings regarding diffusivity indices as a complement to FA [66, 73, 74]. Similarly, few studies have investigated the association of white matter changes in CHR with later transition to psychosis, and their findings are heterogeneous. Bloemen et al. [69] reported decreased FA in the superior temporal lobe and lateral putamen, but increased FA values in the medial temporal lobe in CHR-T compared with CHR-NT, whereas Peters et al. [75] found no differences between groups. On the other hand, Wood et al. [76] reported longer T2 relaxation times (which they used as an indicator of white matter pathology) in CHR-T compared with CHR-NT in the left hippocampal head, and this increase was correlated with psychotic symptom severity. It might be that differences in age (see also further below) and/or time of scanning contribute to these disparate findings: some longitudinal studies failed to find white matter differences between CHR-T and CHR-NT at baseline but reported differences at follow-up [55, 69, 77], while another study [78] reported higher frontal white matter volume at baseline, but more prominent reduction in white matter volume in the left IFOF over time, in CHR-T.

### Multivariate approaches and machine learning

Currently, most neuroimaging studies in psychiatry rely on traditional mass-univariate statistical approaches. While these approaches are useful for detecting features that differentiate between groups, they cannot be used to infer whether an individual patient belongs to a certain group (e.g., CHR-T or CHR-NT) [79]. Therefore, in recent years there has been increased interest for machine-learning methods, which grew out of work in the artificial intelligence field. Machine learning uses a “training” set of existing data to develop mathematical functions that describe complex patterns in the data (i.e., “learn” from experience), which can then be used to make predictions on new data [80]. “Learning” in this context can be supervised or unsupervised: in the first case, the correct classification or outcome is used to train the algorithm, while unsupervised approaches seek to discover structure in the data without any other previous input [81]. Machine-learning models allow inferences at the individual level based on multivariate data with potentially intercorrelated features (a typical example is a spam email filter), and are therefore of great interest in the prediction of psychosis in high-risk patients.

Four studies have used support vector machines, a supervised machine-learning technique, to predict transition to psychosis in CHR patients based on neuroanatomical features [82–85], in partially overlapping patient samples from two CHR centers. In all studies, the pattern that predicted classification was not confined to single regions, but rather extended throughout the brain. Regions reported to contribute most to the decision function included (a) lateral prefrontal [82, 85], medial frontal and cingulate, as well as orbitofrontal areas [82–84]; (b) medial, lateral [84, 85], and inferior temporal including parahippocampal areas [82, 85]; (c) right inferior parietal areas [83, 84]; (d) subcortical structures, most notably the thalamus [82, 85] and basal ganglia [83, 84]; and (e) the cerebellum [83–85].

All of the above studies yielded high classification accuracy (80–88%), with positive predictive values of 78–100% and negative predictive values of 80–90%, suggesting that machine-learning models might be a promising tool in increasing the reliability of transition prediction. However, certain limitations should be kept in mind when interpreting findings. The most important issue is overfitting, which occurs when a statistical model describes noise (i.e., residual variance) in the data rather than genuine effects of interest [79]; this results in very good performance of the model on the observed data, but poor generalizability to unseen data [81]. The high dimensionality of MRI data, with millions of predictor variables for each participant, in combination with modest sample sizes, makes it likely that overfitting may have been an issue in the above studies. Indeed, a recent review of machine-learning techniques in psychiatric neuroimaging studies [79] demonstrated that overall reported accuracy decreased with larger sample sizes. Thus, external validation of machine-learning algorithms in new, independent patient samples is essential for establishing their generalizability and usefulness in clinical practice; so far, there is no external validation for neuroanatomical models of transition to psychosis. Still, the above studies are important in that they show in principle that structural MRI data can be used to support clinical prediction of transition at the individual patient level.

Other implementations of machine-learning models include their use to investigate age-related maturation of brain structure, which has been reported to follow deviant patterns in CHR patients. The North American Prodrome Longitudinal study (NAPLS 2) used structural MRI scans [86] of healthy individuals (*n* = 190) to develop age prediction models, and subsequently calculated the gap between model-predicted age and chronological age in CHR patients (*n* = 380); the Personalized Prognostic Tools for Early Psychosis Management study (PRONIA) implemented a similar approach on neurocognitive performance data of 36 healthy individuals and 48 CHR patients [87]. A larger “brain-age” gap in CHR patients, which was predictive of greater risk of transition to psychosis, was observed in the NAPLS 2 study [86]. The neurocognitive age gap (“CogAGE”) in the PRONIA study was also significantly higher in CHR patients than healthy controls [87], and was associated with increased gray matter volume in frontotemporal areas and diffuse white matter reductions. CogAGE did not predict transition, although this may have been due to sample size issues limiting statistical power.

## Functional neuroimaging

Functional MRI (fMRI) has been widely used in patients with psychotic disorders with the aim of identifying neurobiological substrates of the well-established cognitive impairments associated with the illness, its excellent spatial resolution and noninvasive accessibility substantially contributing to its popularity. Although earlier studies investigated regional activity patterns, recent studies increasingly focus on functional connectivity, i.e., the temporal correlation of activity between different brain areas.

A multitude of fMRI studies in CHR patients have reported abnormalities in brain regional activity and/or functional connectivity during a variety of cognitive tasks [88], including verbal memory and working memory [31, 89–91], verbal fluency [92, 93], social cognition [94–96], as well as in the context of functions more directly implicated in the emergence of psychotic symptoms such as salience processing [97] and evidence gathering [98]. Notably, in many cases, neuroimaging abnormalities were observed even in the absence of differences in behavioral performance. However, these studies are difficult to integrate in a unifying framework given that regional activation profiles and their changes in the high-risk state may vary significantly depending on the assessed function and the specific task or contrast used; for example, CHR patients have been reported to show decreased activity in the middle frontal gyrus in the context of verbal working memory paradigms [31, 89, 90], but increased activity in the same area during verbal fluency tasks [93]. Moreover, very few studies have differences with respect to later transition status; Allen et al. [99] reported increased activation in the prefrontal cortex, midbrain, and left hippocampus as well as increased connectivity between the midbrain and the prefrontal cortex in CHR-T than CHR-NT, but this finding has not yet been replicated. A promising perspective in this field is opened by computational modeling approaches, which use generative models to describe individual task performance and associate it to neuroimaging data [100–102].

Beyond task-based studies, investigations of the resting-state of the brain have been gaining substantial attention in the past two decades, based on findings suggesting that brain activity at rest is not random, and that instead it is organized in functionally meaningful spatiotemporal structures significance [103–107] that affect stimulus processing [105]. Resting-state fMRI studies in CHR patients have reported several disturbances in resting-state networks that are implicated in psychotic disorders, such as decreased coupling within the salience network [72]; dorsostriatal [108, 109], superior temporal [110], and thalamic dysconnectivity; and dysregulated activation of the default mode network [111, 112] including reduced anticorrelations with the task-positive [113, 114] and the salience network [113, 115]. With respect to later psychotic transition, aberrant thalamic connectivity has been reported to be more prominent in CHR-T compared with CHR-NT patients [116].

## Neurotransmitter systems

Positron emission tomography (PET), single photon emission computed tomography, and magnetic resonance spectroscopy (MRS) allow the assessment of neurotransmitter system functionality at the molecular level, and thus are extremely useful in illuminating illness mechanisms, especially with a view to developing pharmacological treatment options. PET and MRS studies in CHR patients have focused on the dopamine and the glutamate system, given the relevance of these two neurotransmitter systems and their interactions for schizophrenia [117].

There are mixed results regarding dopamine availability in CHR; while some studies reported increased dopamine synthesis [99, 118, 119], which was specific to CHR-T in one study [120], other studies failed to find differences in dopamine synthesis capacity [121, 122], D2/D3 receptor availability [123], and synaptic dopamine concentration [124] between CHR and healthy controls. Still, even negative studies in CHR have reported abnormalities such as anomalous associations between dopamine synthesis and verbal memory performance [121] or salience processing [122], as well as reduced positive symptoms following dopamine depletion challenge [124]. Thus, some degree of dopaminergic dysfunction in the high-risk state with potential relevance for later psychotic transition appears plausible. Unfortunately, currently available imaging techniques do not allow differentiation between tonic and phasic aspects of dopaminergic neurotransmission, which are of particular interest given that they are assumed to be differentially affected in psychotic disorders [125].

Markers of glutamatergic neurotransmission such as glutamate and glutamine [generated from glutamate in glia cells [126]] have also been reported to show regional abnormalities in CHR patients, with reduced concentrations in the thalamus [93, 127–129] and elevated concentrations in the prefrontal cortex [129, 130] and the striatum [131]; the latter finding was associated with later transition. With respect to the hippocampus, findings are inconsistent [129, 132, 133]. However, a small study reported a negative association between hippocampal glutamate levels and striatal DOPA uptake in CHR patients (*n* = 16), which was not present in the healthy control sample (*n* = 12) and predicted on a trend level later transition to psychosis [132].

## Electrophysiology

Electrophysiological methods such electroencephalography (EEG) and magnetoencephalography (MEG) have been less prominent than MRI-based techniques in early CHR research. However, advances in analysis techniques and the use of dense array configurations that help to enhance spatial resolution have renewed interest in EEG and MEG in recent years. Advantages of these methods are their excellent temporal resolution, which enables the investigation of the fast dynamics of neuronal interactions occurring on a millisecond time-scale [134], and the possibility to study neuronal network activity in a frequency-specific manner [135–138]. Moreover, emerging evidence suggests that certain neurophysiological measures might reveal coupling patterns that are not captured by fMRI [105]. Therefore, EEG and MEG may be useful complements to structural and functional MRI in disentangling the neurobiological origins of psychotic disorders.

Several neurophysiological biomarkers for schizophrenia have been identified, including early and late event-related potentials (ERP) and other measures of neural oscillatory activity [for reviews, see [139, 140]]. Among these, the best replicated findings concern mismatch negativity. The amplitude of this early preattentive ERP, which is of particular interest because of its association with glutamatergic neurotransmission [140] and the prediction error hypothesis of psychosis [141], is reduced in CHR patients [139, 140]. This reduction has been consistently found to predict transition to psychosis [142–145] with the exception of one study [146] in which, however, statistical power was limited by extremely low transition rates in the CHR group. Another ERP of interest is P300, which is modulated both by the glutamate/GABA [147] and the dopamine system [148]. P300 is also attenuated in CHR [149–153], correlating with gray and white matter abnormalities in these patients [154, 155], and was found to predict future transition in two studies [149, 156]. Gamma-band oscillations are also of potential interest due to their link to the feedback loop between parvalbumin-positive GABAergic interneurons and glutamatergic pyramidal cells [157, 158]. Oscillatory responses in this frequency band are also decreased in CHR [159–161] but have not yet been assessed with respect to later transition; aberrant resting-state gamma-band power has been also reported to predict psychotic transition in CHR patients [162]. Interestingly, EEG synchronization measures in the gamma band are not different between CHR patients and healthy controls, although aberrant synchronization has been observed in lower frequency bands [163–165].

## Discussion

Since the introduction of clinical high-risk criteria in the 1990s, a huge body of structural and functional neuroimaging studies has advanced our understanding of mechanisms associated with risk for, and resilience to, psychotic disorders. Recent studies focus on the utility of structural and functional neuroimaging to improve prediction of outcomes in the individual patient. The findings summarized in the current review testify to the potential of neuroimaging tools as a complement to clinical assessment for quantifying the risk of transition to psychosis in CHR patients. However, several outstanding issues remain, which we address below.

### Data acquisition and preprocessing

Neuroimaging data are collected on special equipment and undergo some extent of data preprocessing. Differences in acquisition parameters or preprocessing protocols may have nontrivial consequences on final results. Common examples include the effect of MRI scanner field strength and manufacturer [166], preprocessing steps such as segmentation, smoothing, and normalization of MRI data [167, 168], or the effects of filtering [169] or wavelet parameters [170] on EEG data. Multicenter studies use harmonized protocols and/or calibration procedures to minimize unwanted variance limiting statistical power [9, 50, 171, 172]. Still, differences in data acquisition and preprocessing may limit comparability between different studies.

### Clinical definitions of CHR state and transition

The diagnosis of both the CHR state and transition to psychosis rely on clinical instruments, which may vary across CHR centers and studies. Although there is substantial diagnostic agreement [173] between the two most widely used clinical instruments for CHR assessment [the *Comprehensive Assessment of At-Risk Mental States* (CAARMS) [5] and the *Structured Interview of Prodromal Syndromes* (SIPS) [174]], they differ with respect to their diagnostic threshold for psychosis, such that some patients categorized as CHR in the CAARMS may meet the criteria for a first psychotic episode in SIPS [173]. Moreover, their equivalence to other instruments [e.g., early recognition inventory ERIraos [175]] has not yet been assessed; neither is it clear how inclusion of CHR patients based on additional criteria [e.g., basic symptoms such as cognitive-perceptive basic symptoms or cognitive disturbances as assessed with the Schizophrenia Proneness Instrument [176, 177], or unspecific risk criteria [178]] may affect findings. An additional issue is that the prognostic accuracy of CHR criteria depends largely on pretest risk, which is higher for help-seeking patients than in the general population [11]. Thus, differences in recruitment setting (specialized early detection center vs. community) may result in substantial differences in participant samples. Finally, an important source of heterogeneity is follow-up duration: because the probability of transition increases with time in the first years from diagnosis [4, 8], studies with shorter follow-up periods are more likely to misclassify patients with later transitions, which might obscure between-group differences.

Another point to consider is that, even using the same diagnostic instrument, the CHR state is not a unitary concept. As detailed in “Introduction”, there are several CHR subgroups, which differ in terms of not only phenomenology but also prognosis [179]. Further research is required to assess whether the differential expression of symptoms in these CHR groups may denote distinct neurobiological substrates.

### Age and developmental issues

A major challenge in early psychosis neuroimaging research is that patients are at an age that is characterized, even in healthy individuals, by major neurodevelopmental changes in the brain. The most notable changes are gray matter loss and cortical thinning [180, 181] and changes in white matter volume and microstructure [182]. These effects conceivably confound results of statistical comparisons and predictive models. Unfortunately, the practice of using age as a covariate of no interest in statistical analyses is likely not sufficient to control for these effects, as changes in gray matter show nonlinear and regionally variable developmental patterns [30], while white matter volume and diffusivity follow different trajectories [182]. ERP biomarkers such as the P300 [183], MMN [184], and resting-state microstates [185] also undergo significant normative changes during development.

Two recent studies showcase the complex interactions of age with neuroanatomy and transition prediction. As detailed above, a larger gap between “brain age” and chronological age and was predictive of greater risk of transition to psychosis, was observed in the NAPLS 2 study; however, this effect was found only in patients diagnosed with CHR status at a young age (i.e., 12–17 years), while it was not present in patients diagnosed at a later age [86]. Another long-term follow-up study [186] reported that surface area decreases in the prefrontal, cingulate, and parahippocampal areas predicted poor symptomatic outcomes in younger, but not in older CHR adolescents. These findings highlight the need to consider patient age when comparing neuroanatomical findings across different studies.

### Power issues

Many neuroimaging studies on CHR patients suffer from relatively small sample sizes, which might lead both to decreased power and increased probability for type II errors. A meta-analysis of studies in patients with established schizophrenia reported that the observed effect sizes with respect to reduction in gray matter structures was in the small to medium range (*d* = 0.22 to *d* = 0.58) [187]. The authors of the meta-analysis calculated that a sample size of *n* = 45 in each group would be necessary to achieve adequate power with an effect size in the low medium range, yet the mean sample size of the studies included in the meta-analysis was only half this value. We observed a similar picture in the single-site studies included in this review, possibly because CHR patients are still underrepresented in clinical referrals for specialized treatment [188]. Moreover, transition rates of ~26% within 3 years from diagnosis [11] result in small CHR-T sample sizes and/or uneven group sizes for comparisons between CHR-T and CHR-NT patients, further reducing statistical power.

### Multiform psychopathology

Most CHR patients suffer from at least one nonpsychotic psychiatric disorder, most often substance-related, affective or anxiety disorders [189, 190], and it has been suggested that APS may be a transdiagnostic indicator of serious psychopathology regardless of transition risk [13]. The presence of multiform psychopathology may contribute additional variance to brain abnormalities observed in CHR patients, but has been rarely considered in neuroimaging studies of the CHR state and transition risk.

### Multimodal—multicenter approaches

Although reviews [191–193] have emphasized superior potential of studies integrating data from different imaging modalities (e.g., functional and structural MRI, or MRI and PET) for obtaining new insights into the neuronal bases of psychotic disorders, such studies are still limited. Moreover, it should be kept in mind that the etiology of psychotic disorders is multifactorial, involving neurobiological and cognitive abnormalities in the context of diverse influencing factors such as genetics, the physical environment, and live events. Thus, models that assess multiple factors and their interactions with each other and time will be informative for theories of symptom emergence, progression, and resolution in CHR patients.

Given the necessity for data-driven, multimodal approaches and adequately powered studies, recently there has been a growing trend toward multicenter research projects. The inclusion of large patient samples and the integration of information across centers and modalities (e.g., neurobiology, genetics, neuropsychology and metacognition, clinical assessment, geo-socio-demographic information) in such projects not only provides better statistical power, but also enables a big-data approach that may contribute to systematic mapping of psychosis development mechanisms and to the development of supplementary diagnostic and/or prognostic tools. Four large multicenter projects are currently implementing such multimodal approaches with a focus on neurobiology and cognition (PRONIA, PSYSCAN), gene-environment interactions (EU-GEI), or combinations of biological data such as neuroimaging, electrophysiology, endocrinology and genomics (NAPLS).

### Limitations and outlook

The present review focused on predictors of psychotic transition in CHR patients. However, several other outcomes are equally relevant for clinical practice. CHR patients who do not develop psychosis suffer from high rates of psychiatric morbidity, persistent subclinical psychotic symptoms, and functional deficits [189, 190, 194, 195]. Several interesting studies have investigated neuroimaging markers of quantitative progression of multi-dimensional symptomatology [50, 186] or poor functional outcome [186, 196–198]. Moreover, given that so far there has been little evidence to favor any one specific intervention for preventing transition to psychosis [199], research into predictors of differential response to different interventions is of great relevance.

On a final note, an important conceptual issue is that the studies we reviewed here define transition to psychosis based on clinical assessment. Hence, the best that can be expected from any of the current predictive models is to replicate clinical diagnostic labels [cf. Viera et al. [81]]. However, as we detailed above, these labels are themselves arbitrary, inconsistent among instruments [200], and do not reflect the complexity of the clinical picture and needs of CHR patients. Implementing bottom-up, multimodal approaches that cut across categorical diagnoses and can help reconceptualize diagnostic classifications represents a greater challenge, and a potential new perspective for neuroimaging in the high-risk state.

## Conclusions

The past two decades of CHR research were exceptionally productive, leading to substantial insights into illness mechanisms leading to psychotic disorders. Still, several issues need to be addressed to achieve clinical applicability, including standardization of clinical definitions and neuroimaging methods, increased focus on generalizability and replicability, and increased attention to contextual factors such as age and comorbidity. The “second wave” of CHR research we are currently experiencing is characterized by large, multimodal/multicenter studies that make use of advances in computing techniques to generate findings that will be useful for individualized prediction in the context of precision psychiatry.
